# *Arabidopsis* VQ motif-containing proteins VQ12 and VQ29 negatively modulate basal defense against *Botrytis cinerea*

**DOI:** 10.1038/srep14185

**Published:** 2015-09-23

**Authors:** Houping Wang, Yanru Hu, Jinjing Pan, Diqiu Yu

**Affiliations:** 1 Key Laboratory of Tropical Forest Ecology, Xishuangbanna Tropical Botanical Garden, Chinese Academy of Sciences, Kunming, Yunnan 650223, China; 2 University of Chinese Academy of Sciences, Beijing 100049, China

## Abstract

*Arabidopsis* VQ motif-containing proteins have recently been demonstrated to interact with several WRKY transcription factors; however, their specific biological functions and the molecular mechanisms underlying their involvement in defense responses remain largely unclear. Here, we showed that two *VQ* genes, *VQ12* and *VQ29*, were highly responsive to the necrotrophic fungal pathogen *Botrytis cinerea*. To characterize their roles in plant defense, we generated *amiR-vq12* transgenic plants by using an artificial miRNA approach to suppress the expression of *VQ12*, and isolated a loss-of-function mutant of *VQ29.* Phenotypic analysis showed that decreasing the expression of *VQ12* and *VQ29* simultaneously rendered the *amiR-vq12 vq29* double mutant plants resistant against *B. cinerea*. Consistently, the *B. cinerea*-induced expression of defense-related *PLANT DEFENSIN1.2 (PDF1.2)* was increased in *amiR-vq12 vq29*. In contrast, constitutively-expressing *VQ12* or *VQ29* confered transgenic plants susceptible to *B. cinerea.* Further investigation revealed that VQ12 and VQ29 physically interacted with themselves and each other to form homodimers and heterodimer. Moreover, expression analysis of *VQ12* and *VQ29* in defense-signaling mutants suggested that they were partially involved in jasmonate (JA)-signaling pathway. Taken together, our study indicates that VQ12 and VQ29 negatively regulate plant basal resistance against *B. cinerea.*

In nature, plants are constantly threatened by various microbial pathogens. To protect themselves against pathogen infection, resisant plants have evolved an effective innate immune system. Upon infection by virulent pathogens, plants detect microbes or pathogen-associated molecular patterns (PAMPs) via transmembrane pattern recognition receptors (PRRs), resulting in PAMP-triggered immunity (PTI)^1^. However, Gram-negative bacterial pathogens can successfully interfere with PTI by secreting effector proteins into plant cells^2^. When pathogens overcome the PTI, plant hosts recognize those effector molecules by specific disease resistance (R) proteins and activate highly efficient immune responses, known as effector-triggered immunity (ETI)^1^.

R proteins-mediated resistance is often associated with activation of the salicylic acid (SA)-signaling pathway that induces a subset of *PATHOGENESIS-RELATED GENE (PR)* genes^3^. *Arabidopsis* mutants deficient in SA biosynthesis (*e.g., sid2*) or responsiveness (*e.g., npr1*) are compromised to establish both basal defense and systemic acquired resistance^4^,^5^. Besides SA, ethylene (ET) and JA also play crucial roles in plant defense responses to pathogen attack^6^,^7^. The ET signal transduction components ETHYLENE INSENSITIVE2 (EIN2), EIN3 and EIN3-Like1 (EIL1) function as critical positive regulators of plant disease resistance toward necrotrophic pathogens^8^,^9^,^10^,^11^. In addition, the JA receptor CORONATINE INSENSITIVE1 (COI1) also positively regulates plant tolerance against necrotrophic pathogens^12^,^13^,^14^.

The *Arabidopsis* WRKY transcription factor family comprises 74 members which are subdivided into three major structural groups^15^,^16^. Accumulating evidence has indicated that WRKY proteins act as both positive and negative regulators in modulating plant defense responses^17^,^18^,^19^. For example, WRKY33 positively regulates plant resistance to the necrotrophic fungal pathogens *Botrytis cinerea* and *Alternaria brassicicola*^20^. Disruption of the structurally related WRKY46, WRKY70 and WRKY53 compromised plant basal defense against biotrophic pathogen *Pseudomonas syringae*^21^. In contrast, several WRKY family members negatively modulate plant pathogen resistance. For instance, the evolutionarily related WRKY18, WRKY40 and WRKY60 function as negative regulators of plant resistance against *P. syringae*^22^. Mutations of *WRKY11* and *WRKY17* resulted in increased defense-related gene expression and enhanced basal defense to *P. syringae*^23^.

Recently, several WRKY transcription factors have been found to physically interact with a class of novel proteins, defined as VQ proteins, to regulate various physiological processes^24^,^25^,^26^,^27^,^28^,^29^. The name of VQ proteins are derived from the FxxxVQxxTG motif, a conserved amino acid region shared by all members of this family in *Arabidopsis*^27^,^30^. Increasing studies have demonstrated that VQ proteins play crucial roles in modulating plant defense responses. For example, VQ21 functions as one substrate of MAP kinase 4 (MPK4) and is involved in MPK4-mediated resistance^24^,^31^,^32^. The structurally related VQ16 and VQ23, previously identified as sigma factor binding proteins, redundantly regulate plant defense response against *B. cinerea*^26^,^30^,^33^. In addition, the JA-associated VQ protein VQ22 modulates JA-mediated defense responses^34^. Besides defense responses, VQ proteins are also involved in abiotic stress responses. Our previous study revealed that VQ9 interacts with WRKY8 and participates in plant salinity tolerance^28^. In an earlier study, VQ15 was identified as a calmodulin (CaM)-binding protein that affects osmotic stress tolerance^35^. Moreover, several developmental processes are also modulated by VQ proteins; for example, VQ14 was reported to play an essential role in seed development^25^,^36^. Very recently, Li *et al.* showed that VQ29 acts as a negative transcriptional regulator of light-mediated inhibition of hypocotyl elongation by interacting with PHYTOCHROME-INTERACTING FACTOR1 (PIF1)^37^.

Cheng *et al.* showed that a number of *Arabidopsis VQ* genes were responsive to plant defense signals^27^. However, the specific biological functions of *VQ* genes and the exact mechanisms underlying their involvement in defense responses remain largely unknown. To further clarify the functions of *Arabidopsis VQ* genes in plant defense, we chose *VQ12* and *VQ29* for further investigation. *VQ12* and *VQ29* were strongly induced by JA treatment and *B. cinerea* infection; and the proteins encoding by *VQ12* and *VQ29* were exclusively localized in the nucleus. Phenotypic analysis indicated that the resistance of *vq29* mutant plants to *B. cinerea* was enhanced compared with that of wild type. Moreover, decreasing the expression of *VQ12* and *VQ29* simultaneously conferred the *amiR-vq12 vq29* double mutant plants even greater resistance against *B. cinerea.* In contrast, the transgenic plants overexpressing *VQ12* or *VQ29* were much more susceptible to *B. cinerea*. Further investigation revealed that VQ12 and VQ29 physically interacted with themselves and each other to form homodimers and heterodimer. Taken together, our results provide evidence that VQ12 and VQ29 negatively regulate plant basal resistance against *B. cinerea.*

## Results

### *VQ12* and *VQ29* genes are strongly responsive to *B. cinerea*

**Figure 1:**
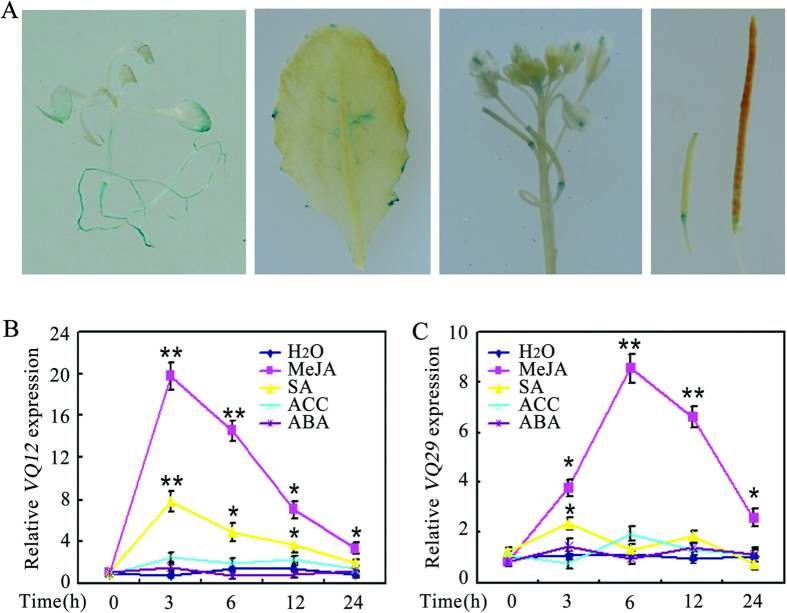
Analysis of *VQ12* and *VQ29* expression. (**A)** GUS staining of whole eight-day-old *Arabidopsis* transgenic seedlings and various tissues expressing the *GUS* reporter gene under the control of the *VQ12* promoter. **(B,C)** qRT-PCR analysis of *VQ12* (**B**) and *VQ29* (**C**) expression in response to defense-related hormones. Total RNA was extracted from thirty-day-old wild-type plants at given times after spraying with H_2_O, MeJA (100 μM), SA (1 mM), ACC (2 mM) or ABA (100 μM). Error bars indicate SD from three independent RNA extracts; statistics by Student’s t test; *p < 0.05; **p < 0.01.

**Figure 2:**
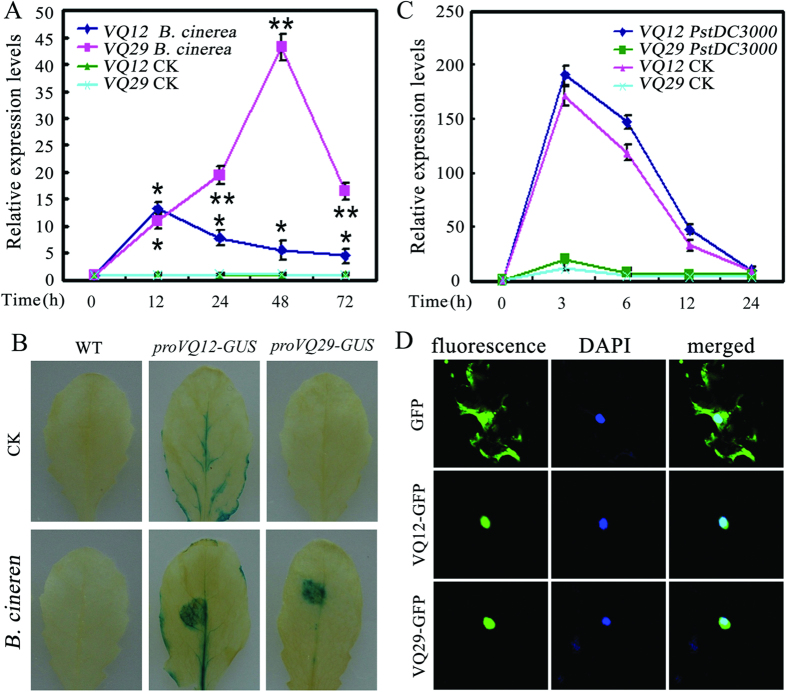
Pathogen-induced expression and subcellular localization of VQ12 and VQ29. **(A)** qRT-PCR analysis of *VQ12* and *VQ29* expression in response to *B. cinerea*. Total RNA was extracted from thirty-day-old wild-type plants (WT) at given times after spraying with *B. cinere*a or Sabouraud maltose broth (SMB) buffer (CK). Error bars indicate SD from three independent RNA extracts. **(B)** GUS staining of WT, *proVQ12-GUS* and *proVQ29-GUS* leaves treated with *B. cinere*a or SMB buffer (CK) for 24 h. **(C)** qRT-PCR analysis of *VQ12* and *VQ29* expression in response to *P. syringae*. Total RNA was extracted from thirty-day-old wild type at given times after infiltration with a suspension of *Pst*DC3000 or MgCl_2_ (CK). Error bars indicate SD from three independent RNA extracts; statistics by Student’s t test; *p < 0.05; **p < 0.01. **(D)** Subcellular localization of VQ12 and VQ29 proteins. VQ12-GFP, VQ29-GFP and free GFP were transformed into *N. benthamiana* epidermal cells. DAPI staining marks the nucleus.

*Arabidopsis VQ12* (AT2G22880) and *VQ29* (AT4G37710) encode two VQ motif-containing proteins with 114 and 123 amino acids, respectively^27^. To characterize their biological functions, we generated homozygous T3 lines of *promoterVQ12:GUS* and *promoterVQ29:GUS* transgenic plants. β-Glucuronidase (GUS) staining showed that *VQ12* was mainly expressed in the root, leaf, hypocotyl, and silique base (Fig. 1A), which is similar to the basic expression pattern of *VQ29*^37^. To determine the expression profiles of *VQ12* and *VQ29* more precisely, we further analyzed their induced expression in response to various defense-related hormones. As shown in Fig. 1B, expression of *VQ12* was induced by methyl jasmonate (MeJA) and SA, but not by abscisic acid (ABA) and 1-aminocyclopropane-1-carboxylate (ACC). Similarly, the expression level of *VQ29* was also upregulated by MeJA treatment (Fig. 1C). Further quantitative RT-PCR (qRT-PCR) analysis showed that the *VQ12* and *VQ29* transcripts accumulated high levels in *B. cinerea-*infected plants (Fig. 2A); and these results were confirmed by GUS staining, as high GUS activities were detected in leaves of *promoterVQ12:GUS* and *promoterVQ29:GUS* transgenic plants after *B. cinerea* infection (Fig. 2B). However, the expression of *VQ12* and *VQ29* was not responsive to *Pst*DC3000 infection (Fig. 2C). Together, these results indicate that *VQ12* and *VQ29* mainly respond to MeJA and *B. cinerea*-infection and may be involved in disease resistance against *B. cinerea.*

To determine the properties of VQ12 and VQ29 in more detail, we next analyzed their subcellular localizations. The full-length VQ12 and VQ29 were fused to the green fluorescent protein (GFP) protein under the control of the *Cauliflower mosaic virus* (CaMV) 35S promoter and these constructs were transiently expressed in leaves of tobacco (*Nicotiana benthamiana*). As shown in Fig. 2D, the transiently expressed VQ12-GFP and VQ29-GFP fused proteins displayed fluorescence exclusively in the nucleus, as revealed by 4’,6-diamidino-2-phenylindole (DAPI) staining. In the control, free GFP was observed in both the cytoplasm and the nucleus (Fig. 2D). These observations indicate that VQ12 and VQ29 are nuclear proteins and may function in the nucleus.

### Decreasing the expression of *VQ12* and *VQ29* simultaneously enhances plant resistance against *B. cinerea*

To characterize the function of VQ12 in plant defense against *B. cinerea*, we generated *amiR-vq12* transgenic plants by using an artificial miRNA approach to repress *VQ12* expression^38^. qRT-PCR analysis showed that the transcripts of *VQ12* in *amiR-vq12* transgenic lines 5 and 7 (*amiR-vq12*–*5* and *amiR-vq12–7*) were reduced compared with those in wild-type plants with or without MeJA treatment (Supplementary Figure S1). We also identified a T-DNA insertion mutant for *VQ29* from the Salk T-DNA population. *vq29* (Salk_061438) mutant harbors a T-DNA insertion in the promoter region (−136 bp from the translation start site) of *VQ29* (Supplementary Figure S2)^37^. Further examination indicated that the expression of *VQ29* was significantly decreased in *vq29* compared with that in wild type (Supplementary Figure S2)^37^. To clarify the possible functional cooperation between VQ12 and VQ29, we generated *amiR-vq12 vq29* double mutant plants by crossing *amiR-vq12–5* with *vq29*.

**Figure 3:**
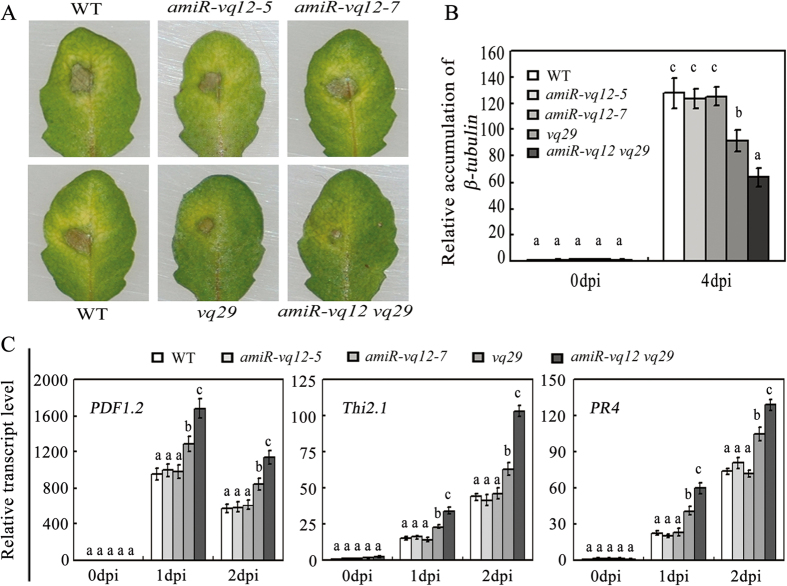
Responses of *vq29*, *amiR-vq12* and *amiR-vq12 vq29* plants to *B. cinerea.* **(A)** Leaves of various plants were drop-inoculated with *B. cinerea* spores (2 × 10^5^ spores/ml). The disease symptoms were photographed at 3dpi. **(B)** Accumulation of *B. cinerea β-tubulin.* Total RNA was isolated from inoculated plants at 0 and 4 dpi, and the expression levels of *β-tubulin* were analyzed using *B. cinerea β-tubulin* gene-specific primers. **(C)** qRT-PCR analysis of *PDF1.2, Thi2.1* and *PR4* expression levels. Total RNAs were extracted from *B. cinerea*-inoculated leaves harvested at 0, 1, and 2 dpi. In (**B**) and (**C**) values are mean SE (n = 3 experiments), and different letters above columns indicate significant differences based on Tukey’s test (P < 0.05).

To determine the possible functions of VQ12 and VQ29 in plant defense, we analyzed the performance of *amiR-vq12* lines, *vq29* and *amiR-vq12 vq29* in response to *B. cinerea* infection. Thirty-day-old plants were challenged with a *B. cinerea* spore suspension (5 × 10^5^ spores/ml). As shown in Fig. 3A, no significant difference between the *amiR-vq12* lines and wild type in disease symptom development was observed. However, following infection with *B. cinerea*, the *vq29* mutant plants showed reduced disease symptom with restricted disease lesions in comparison with wild-type plants (Fig. 3A). Moreover, the *amiR-vq12 vq29* double mutant plants were substantially more resistant against *B. cinerea* compared with *vq29* and wild type (Fig. 3A). To confirm these disease symptoms, we quantified the biomass of the infecting pathogen by examining the transcripts of *β-tubulin* gene of *B. cinerea* in inoculated plants. As shown in Fig. 3B, lower levels of *β-tubulin* mRNA of *B. cinerea* was detected in *vq29* and *amiR-vq12 vq29* plants at 4 d after post-inoculation (dpi). Since *vq29* and *amiR-vq12 vq29* plants acquired more resistance against *B. cinerea*, the pathogen-induced expression of *PDF1.2*, the antifungal gene *THIONIN2.1* (*Thi2.1*), and the pathogenesis response gene *PR4*, were also analyzed^34^,^39^,^40^. Compared to those in wild-type plants, *PDF1.2, Thi2.1* and *PR4* transcripts were increased in *vq29* and *amiR-vq12 vq29* plants after *B. cinerea* infection (Fig. 3C). Taken together, these results indicate that decreasing the expression of *VQ12* and *VQ29* simultaneously enhances plant resistance against *B. cinerea* infection.

### Overexpression of *VQ12* or *VQ29* confers plants susceptible to *B. cinerea*

**Figure 4:**
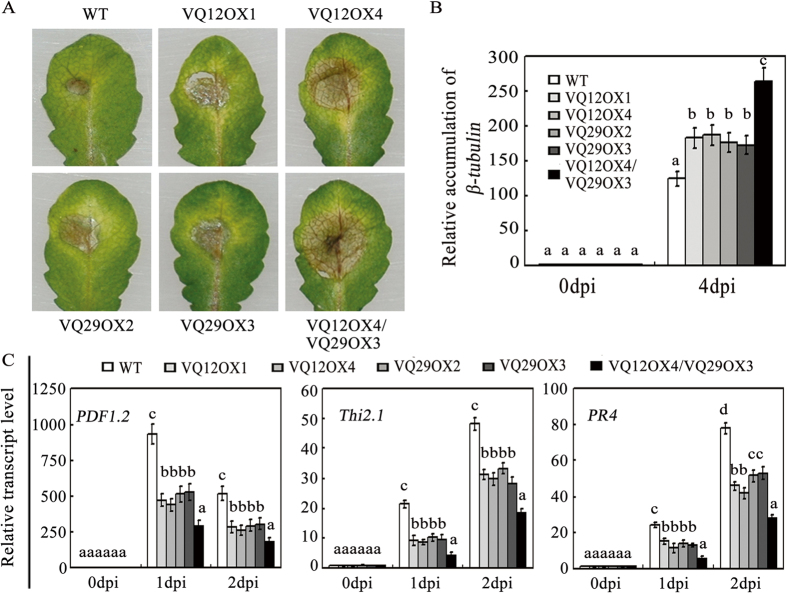
Responses of transgenic plants overexpressing *VQ12* or *VQ29* to *B. cinere*a. **(A)** Leaves of various transgenic plants were drop-inoculated with *B. cinerea* spores, and the disease symptoms were photographed at 2 dpi. **(B)** Accumulation of *B. cinerea β-tubulin.* Total RNA was isolated from inoculated plants at 0 and 4 dpi, and the expression levels of *β-tubulin* were analyzed using *B. cinerea β-tubulin* gene-specific primers. **(C)** qRT-PCR analysis of *PDF1.2, Thi2.1* and *PR4* expression levels. Total RNAs were extracted from *B. cinerea*-inoculated leaves harvested at 0, 1, and 2 dpi. In (**B**) and (**C**) values are mean SE (n = 3 experiments), and different letters above columns indicate significant differences based on Tukey’s test (P < 0.05).

To further investigate the roles of VQ12 and VQ29 in plant defense, we generated transgenic plants overexpressing *VQ12* or *VQ29* under the control of the CaMV 35S promoter. qRT-PCR analysis showed that several overexpression lines constitutively expressed elevated levels of *VQ12* or *VQ29* transcripts even without any treatment (Supplementary Figure S3). Two lines of VQ12 (VQ12OX1 and VQ12OX4) and two lines of VQ29 (VQ29OX2 and VQ29OX3) were selected for further study (Supplementary Figure S3). The F_2_ progeny of those homozygous transgenic plants showed the same morphology as wild-type plants under normal growth conditions for thirty days. Then, we analyzed the performances of those overexpression plants in response to *B. cinerea* infection. After *B. cinerea* inoculation, those overexpression plants showed enhanced susceptibility to this fungal pathogen with more obvious leaf decay and greater accumulation of fungal biomass, compared to wild-type plants (Fig. 4A,B). Moreover, the plants constitutively expressing both VQ12 and VQ29 (VQ12OX4/VQ29OX3) simultaneously exhibited even more severe disease symptoms than VQ12OX4, VQ29OX3 and wild type (Fig. 4A,B). Consistent with the disease symptoms, the *B. cinerea* induced-expression levels of *PDF1.2, Thi2.1* and *PR4* genes in *VQ12* or *VQ29* over-expressing plants were reduced compared with those in wild type (Fig. 4C). Thus, overexpression of *VQ12* and/or *VQ29* increases plant susceptibility to the fungal pathogen *B. cinerea.*

### VQ12 and VQ29 physically interact to form heterodimer and homodimers

**Figure 5:**
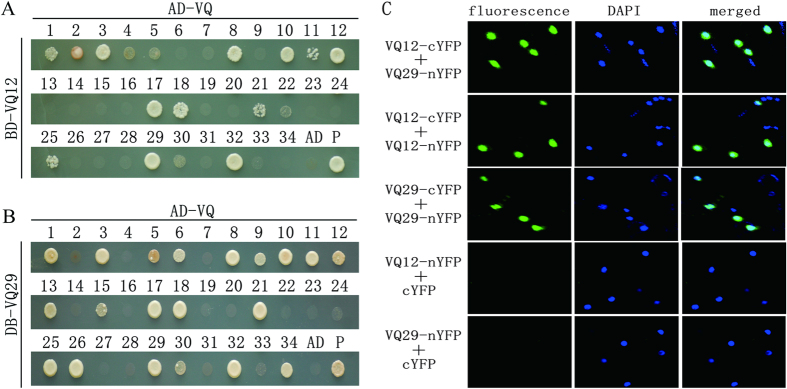
Physical interactions between VQ proteins. **(A,B)** Yeast two-hybrid analysis of VQ12 (**A**) and VQ29 (**B**) interactions with all VQ proteins. Interaction was indicated by the ability of cells to grow on selective media lacking Leu/Trp/His/Ade and containing 10 mM 3-aminotriazole. WRKY33 interactions with VQ12 (**A**) and VQ29 (**B**) were used as positive controls (P). **(C)** BiFC assay showing the fluorescence complementation of the C-terminal part of YFP fused with VQ12 or VQ29 and the N-terminal part of YFP fused with VQ12 or VQ29. DAPI staining marks the nucleus.

To understand how VQ12 and VQ29 modulate plant resistance against *B. cinerea*, we employed the yeast two-hybrid system to identify their potentially interacting partners. As the full-length VQ12 and VQ29 proteins showed strong transcriptional activation activities, the VQ12 and VQ29 proteins with deleted activation domains (deleted amino acids 1 to 22 for VQ12 and deleted amino acids 1 to 21 for VQ29) were fused to the Gal4 DNA-binding domain of the bait vector (BD-VQ12 and BD-VQ29). After screening, more than 30 independent colonies were isolated. Among these positive clones, clones encoding VQ proteins were frequently represented. To confirm their physical interactions, the full-length coding sequences of all 34 *Arabidopsis* VQ proteins were cloned and introduced into the prey vector (AD-VQ). The BD-VQ12 or BD-VQ29 and AD-VQ plasmids were co-transformed into yeast. As shown in Fig. 5A, VQ12 strongly interacted with eight VQ proteins (VQ3, VQ8, VQ10, VQ12, VQ17, VQ18, VQ29, and VQ32) and slightly interacted with ten other VQ proteins (VQ1, VQ2, VQ4, VQ5, VQ11, VQ21, VQ22, VQ25, and VQ30) in yeast. Similarly, VQ29 strongly interacted with seventeen VQ proteins (VQ1, VQ3, VQ6, VQ8, VQ9, VQ10, VQ11, VQ12, VQ13, VQ17, VQ18, VQ21 VQ25, VQ26, VQ29, VQ32, and VQ34), and slightly interacted with three other VQ proteins (VQ5, VQ15, and VQ30) (Fig. 5B). To determine whether VQ12 and VQ29 interact with themselves or each other in plant cells, we used BiFC assay for further analysis. Full-length VQ12 and VQ29 proteins were fused to the N-terminal region of the yellow fluorescent protein (nYFP) and C-terminal region of YFP (cYFP). When VQ12-cYFP was co-infiltrated with VQ12-nYFP or VQ29-nYFP in tobacco leaves, strong YFP fluorescence was detected in nuclei (Fig. 5C). Similarly, tobacco leaves co-expressing VQ29-cYFP and VQ29-nYFP also showed strong YFP fluorescence (Fig. 5C). We did not detect any fluorescence in all negative controls (Supplementary Figure S4). These results indicate that VQ12 and VQ29 physically interact to form heterodimer and homodimers.

### C-terminal parts are required for VQ12-VQ29 physical interaction

**Figure 6:**
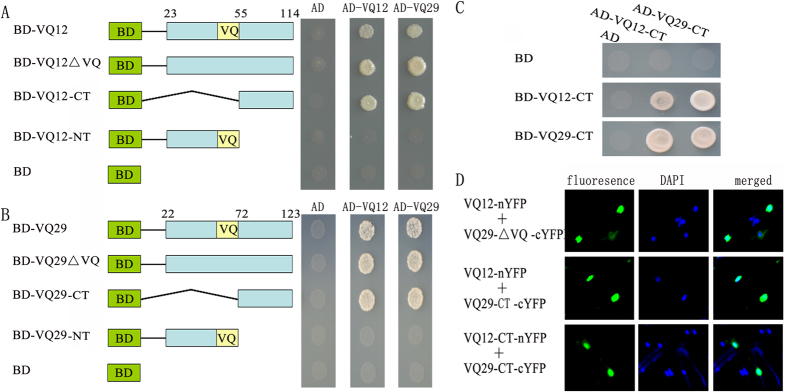
C-terminal parts are required for VQ proteins to form hetero- or homo-dimers. **(A)** The C-terminal part of VQ12 was required for interaction with itself or VQ29. **(B)** The C-terminal part of VQ29 was essential for interaction with itself or VQ12. **(C)** The C-terminal fragments of VQ12 and VQ29 are sufficient for their interactions. Interactions were indicated by the ability of cells to grow on selective media lacking Leu/Trp/His/Ade and containing 10 mM 3-aminotriazole. The empty pGADT7 prey vector was used as a negative control. **(D)** BiFC analyses.

To characterize which domain of VQ12 or VQ29 is responsible for the interactions with themselves or each other, VQ12 and VQ29 were divided into N-terminal parts containing the VQ motif and C-terminal portions (Fig. 6). Moreover, we also mutated the amino acids (LVQR) in VQ motifs of VQ12 and VQ29 to EDLE (Fig. 6). The directed yeast two-hybrid analysis showed that deleting the N-terminal residues or replacing the VQ motifs of VQ12 and VQ29 did not affect the physical interactions with themselves or each other (Fig. 6A,B). However, deletion of the C-terminal parts of VQ12 or VQ29 eliminated those interactions (Fig. 6A,B). To further characterize whether the C-terminal fragments are sufficient for their interactions, the C-terminal portions of VQ12 and VQ29 were cloned and introduced into the prey vector. The yeast two-hybrid results show that the C-terminal part of VQ12 or VQ29 interacted with themselves or each other (Fig. 6C). To further determine the interaction from yeast two-hybrid assay, we used the BiFC assay to analyze their physical interactions. The BiFc results also indicate that the C-terminal parts of VQ12 and VQ29 interacted with themselves or each other in plant cells (Fig. 6D and Supplementary Figure S5).

We further re-examined the properties of different domains of VQ12 and VQ29 in more detail by analyzing their sub-cellular localization. Different domains of VQ12 and VQ29 were fused to the GFP protein and these fused proteins were transiently expressed in leaves of tobacco. As shown in Supplementary Figure S6, the transiently expressed VQ12-CT-GFP, VQ29-CT-GFP, VQ12△VQ-GFP, and VQ29△VQ-GFP fusion proteins were exclusively localized in the nucleus. These results were consistent with their abilities to interact with themselves or each other in nuclei. However, the VQ12-NT-GFP and VQ29-NT-GFP were localized in both the nucleus and the cytoplasm (Supplementary Figure S6). These results indicate that the C-terminal parts of VQ12 and VQ29 are critical for their nuclear localizations.

### VQ12 and VQ29 are partially involved in JA-mediated signaling pathway

**Figure 7:**
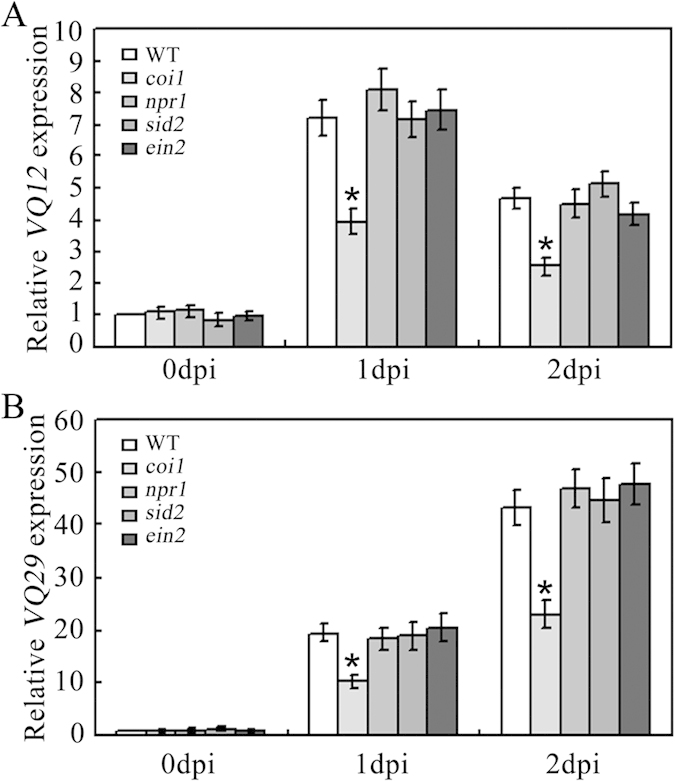
Partial involvement of VQ12 and VQ29 in JA-signaling pathway. qRT-PCR analysis of *VQ12* (**A**) and *VQ29* (**B**) expression in response to *B. cinerea* in wild type (WT) and various defense-related mutants. Total RNA was extracted from thirty-day-old wild-type or mutant plants at 0, 1 and 2 dpi. Error bars indicate SD from three independent RNA extracts; statistics by Student’s t test; *p < 0.05.

In *Arabidopsis*, several signaling pathways are involved in defense responses, such as the SA-, ET- and JA-signaling pathways. To analyze which pathway *VQ12* and *VQ29* are involved in, we monitored their expression in various defense-related mutants, including *sid2* (SA-biosynthesis mutant), *npr1* (SA-signaling mutant), *coi1* (JA-signaling mutant) and *ein2* (ET-signaling mutant)^4^,^8^,^12^,^14^,^41^. Before infection with *B. cinerea*, the basic expression levels of *VQ12* and *VQ29* were not affected in those mutants (Fig. 7A,B). When inoculated with *B. cinerea*, the expression levels of *VQ12* and *VQ29* were strongly induced in *sid2*, *npr1* and *ein2* mutants, as those in wild-type plants. However, their induced-expression levels were remarkably reduced in the *coi1* mutant (Fig. 7A,B). As a negative control, their expression levels in *sid2* and *npr1* mutants after *Pst*DC3000 infection were not altered compared with those in wild type (Supplementary Figure S7). Those observations indicate that the *B. cinerea*-induced expression of *VQ12* and *VQ29* may be partially dependent on the JA-signaling pathway.

## Discussion

Recently, several studies demonstrated that members of *VQ* family are responsive to pathogen and/or defense-related hormones. For example, Cheng *et al.* showed that a number of *Arabidopsis VQ* genes responded to SA treatment and/or the bacterial pathogen *Pst*DC3000 infection^27^. Several *VQ* genes of *Arabidopsis* and tomato were also strongly induced by fungal pathogen infection and/or treatment with fungal elicitors^26^,^42^. In addition, Kim *et al.* reported that the expression of several rice *VQ* genes was up-regulated by the bacterial pathogen *Xanthomonas oryzae pv. Oryzae*^43^. These results suggested that VQ proteins may play regulatory roles in plant defense responses; however, direct evidence for their associations with defense responses remains largely limited. Investigating specific fuctions of VQ proteins and VQ-mediated signaling pathways may provide new insight on the molecular basis of plant defense responses.

In this study, we found that *VQ12* and *VQ29* were highly responsive to JA treatment and *B. cinerea* infection, but not responsive to *Pst*DC3000 infection (Figs 1 and 2). However, in Fig. 2C, it appears that *VQ12* expression is induced by infiltration with MgCl_2_, indicating that the expression of VQ12 may also be responsive to wounding. Interestingly, accumulating evidence has indicated that some defense-related genes are also wounding-induced. For example, the *VQ* family member *VQ22* which modulates JA-mediated defense responses is also responsive to wounding treatments^34^. In addition, our earlier study showed that the defense-associated *WRKY8* is also strongly induced by wounding^44^. These results suggest that there may be some associations between wounding and defense responses.

Further phenotypic analysis showed that VQ12 and VQ29 negatively regulate plant resistance against *B. cinerea*, as the *amiR-vq12 vq29* double mutant plants displayed greater resistance compared with *vq29* single mutant and wild type (Fig. 3). Consistent with those findings, the induced expression levels of defense-related *PDF1.2, Thi2.1* and *PR4* genes were increased in *B. cinerea*-infected *amiR-vq12 vq29*, compared with that in wild type (Fig. 3). In contrast, transgenic plants constitutively-expressing *VQ12* or *VQ29* were much more susceptible to *B. cinerea* (Fig. 4). Taken together, these JA-responsive VQ12 and VQ29 proteins function as negative regulators in plant basal defense against *B. cinerea*.

Similarly, several other reported VQ proteins, such as VQ5, VQ20, VQ21 and VQ22, also negatively mediate plant basal defense against *B. cinerea*^27^,^32^,^34^. For example, Hu *et al.* showed that decreasing the expression of *VQ22* gene enhanced plant resistance against *B. cinerea*^34^. However, Lai *et al.* showed that two structurally related VQ16 and VQ23 positively regulate plant defense responses against *B. cinerea*^26^. This means that they function as positive regulators of plant defense against *B. cinerea*. Moreover, several VQ proteins were shown to be involved in plant resistance against herbivorous insects and/or the biotrophic pathogen *P. syringae*^24^,^27^,^29^,^34^. The multiple roles of VQ proteins in plant defense may suggest that the signal transduction of defense responses require tight regulation and fine-tuning. It’s possible that VQ proteins play crucial roles in maintaining proper balance of different signaling pathways, resulting in appropriate tolerance against pathogens and/or insects parasitism while minimizing detrimental effects on plant growth. However, the exact molecular mechanisms underlying their involvement in defense responses are still largely unclear. Further researches are required to identify their putative interacting proteins and illustrate the signaling pathways they are involved in.

Over the past several years, several proteins have been reported to physically interact with VQ family members, including WRKYs, MPKs and PIF1^24^,^25^,^27^,^28^,^29^,^37^. For example, Cheng *et al.* revealed that VQ proteins interacted with group I and IIc WRKY transcription factors and their VQ motifs were essential for those interactions^27^. Interestingly, accumulating evidence has demonstrated that WRKY transcription factors play crucial roles in regulating plant defense responses^17^. Therefore, the physical interactions between WRKY factors and VQ proteins may provide an important mechanism for regulating plant defense responses. In this study, we further found that VQ12 and VQ29 physically interacted with themselves and several other VQ members to form homodimers and heterodimer (Fig. 5). Further investigation revealed that the C-terminal fragment but not the VQ motif was required for VQ–VQ protein interactions (Fig. 6). Thus, it’s possible that VQ12 and VQ29 interact with themselves or each other via their C-terminal parts, while interact with WRKY33 via the VQ motif to form a big protein complex to mediate plant defense responses against *B. cinerea*. Nevertheless, future experiments are needed to analyze the regulatory effect of VQ12 and/or VQ29 on WRKY33 factor in modulating defense responses.

The phytohormone JA, as a crucial defense signal, positively regulates plant resistance against necrotrophic pathogens and herbivorous insects. The JA receptor COI1 has been shown to play critical roles in plant defense responses, as its mutations result in enhanced susceptibility to necrotrophic pathogens and herbivorous insects^12^,^13^,^14^. In this study, we observed that, after infection with *B. cinerea* pathogen, the *coi1* mutant plants showed decreased expression of *VQ12* and *VQ29*, compared to wild type (Fig. 7). This observation suggested that the *B. cinerea*-induced expression of *VQ12* and *VQ29* is involved in JA-mediated signaling pathway. Similarly, Hu *et al.* showed that the induced expression of *VQ22* was also associated with JA signaling^34^. Interestingly, further analysis in their study revealed that the regulation or degradation of VQ22 protein was also involved in JA signaling. Therefore, JA signaling plays dual roles to regulate VQ22 during defense responses. It’s interesting to illustrate the exact molecular mechanisms underlying the regulation of VQ members by JA signaling in future studies.

## Materials and Methods

### Materials and *Arabidopsis* growth conditions

The *vq29* (Salk_061438) mutant and all transgenic plants used in this study were derived from the *Arabidopsis* Columbia (Col-0) ecotype. *Arabidopsis* plants were grown in growth chambers at 22°C with a 10-h light/14-h dark photoperiod. The mutant lines *npr1*, *sid2*, *coi1* and *ein2* were kindly gifted by Dr. Zhixiang Chen (Purdue University, USA). The plant hormones MeJA, SA, ABA and ACC were purchased from Sigma-Aldrich. Taq DNA polymerase was purchased from TaKaRa Biotechnology Co. Ltd. Other common chemicals were obtained from Shanghai Sangon Biotechnology Co. Ltd.

### Pathogen infection and induction treatments

*Pst*DC3000 infection was performed as described in^21^. Collection of *B. cinerea* spores and plant inoculation was performed as described previously^44^. For GUS staining and single-leaf drop inoculations, a single 3-μl drop of a suspension of 2 × 10^5^ spores/ml in Sabouraud maltose broth (SMB) buffer was placed on each leaf. Induction treatments with the plant hormones MeJA, SA and ACC were performed as described in Chen *et al.*^44^.

### Subcellular localization

Full-length VQ12 and VQ29 fused to GFP were cloned into the pOCA30 binary vector, downstream of the CaMV 35S promoter. The constructs were then transformed into *Agrobacterium tumefaciens* strain EHA105. For transient expression, *N. benthamiana* leaves were infiltrated with the bacterial cell suspensions (optical density at 600 nm [OD_600_] = 0.05, 10 mM MES, 10 mM MgCl_2_, and 100 mM acetosyringone). After infiltration, the plants were incubated at 24 °C for 4 h, and then the infected leaves were sectioned for observation. GFP and DAPI fluorescence were observed under a confocal laser scanning microscope (Olympus, Japan).

### Identification of T-DNA insertion mutants and construction of transgenic plants

The *vq29* mutant contains a T-DNA insertion in the promoter of *VQ29* gene. We confirmed the T-DNA insertion by PCR using a combination of a T-DNA border primer (5′-AAACGTCCGCAATGTGTTAT-3′) and a gene-specific primer (vq29m-A: 5′-GATCAAGGAATCCGATTAGATTCA-3′; vq29m-B: 5′- GAAGGTTGTTTACGTGTCGAATG-3′). The insertion mutant was further confirmed by qRT-PCR. To generate the *VQ12* and *VQ29* overexpression constructs, the full-length *VQ12* and *VQ29* gene coding sequences were PCR-amplified from *Arabidopsis* gDNA and cloned into the pOCA30 vector in the sense orientation behind the CaMV 35S promoter^45^. To suppress *VQ12* expression we used an artificial miRNA approach as described in Liang *et al.*^38^. An *amiR-vq12* sequence was designed using WMD3 (http://wmd3.weigelworld.org), with an *AthmiR319a* backbone to drive its expression^38^,^46^. Then, the *amiR-vq12* precursor was cloned into the pOCA30 vector. All constructs were transformed into *Arabidopsis* plants using the *Agrobacterium*-mediated flower dip method. Homozygous transgenic *amiR-vq12*–5 line were crossed with *vq29* to generate *amiR-vq12 vq29* homozygous plants. VQ12OX4/VQ29OX3 plants were generated through genetic crosses of VQ12OX4 and VQ29OX3 homozygous transgenic lines.

### RNA extraction and qRT-PCR

Total RNA was extracted using the Trizol reagent (Invitrogen). One microgram of DNase-treated RNA was reverse-transcribed in a 20 μl reaction mixture using Superscript II (Invitrogen). After the reaction, 1 μl cDNA was subjected to qRT-PCR using a SYBR Premix Ex Taq kit (Takara) on a Roche LightCycler 480 real-time PCR machine. At least three independent biological samples were used for qRT-PCR analysis for each reported result. *ACTIN2* (AT3G18780) was used as an internal control. The gene-specific primers for qRT-PCR are listed in Supplementary Table S1.

### GUS reporter analysis

The putative promoters of *VQ12* and *VQ29* were amplified from genomic DNA using gene-specific primers (Supplementary Table S1). The constructs *proVQ12:GUS* and *proVQ29:GUS* were cloned into the pOCA28 binary vector. Transgenic plants were subjected to GUS staining as described in Chen *et al.*^44^.

### Yeast two-hybrid screening and confirmation

The truncated VQ12 and VQ29 CDSs were cloned into the bait vector pGBKT7 and then transformed into the yeast strain Y2HGold (Clontech). The cDNA library was obtained from Clontech (Catalog number 630487). Yeast two-hybrid screening was performed as described in Hu *et al.*^47^. To confirm protein–protein interactions, full-length CDSs of all 34 VQ proteins were cloned into the prey vector pGADT7. Primers used for amplifying these fragments for yeast two-hybrid assays are listed in Supplementary Table S1.

### Bimolecular fluorescence complementation (BiFC) assay

The cDNA sequences for the N-terminal 173-amino acid eYFP (N-YFP) and C-terminal 64-amino acid (C-YFP) fragments were cloned into pFGC5941 to generate pFGC-nYFP and pFGC-cYFP, respectively^28^. Full-length coding sequences of *VQ12* and *VQ29* were cloned into pFGC-nYFP to generate N-terminal in-frame fusions with N-YFP, while *VQ12* and *VQ29* CDSs were inserted into pFGC-C-YFP to form C-terminal in-frame fusions with C-YFP. The plasmids were transformed into *A. tumefaciens* strain EHA105, and infiltration of *N. benthamiana* was performed as described previously^48^. Infected leaves were analyzed at 48 h after infiltration. YFP fluorescence and DAPI staining were observed under a confocal laser scanning microscope (Olympus, Japan).

## Additional Information

**Accession codes:** *Arabidopsis* Genome Initiative numbers for the genes discussed in this article are as follows: *VQ12* (AT2G22880), *VQ29* (AT4G37710), *PDF1.2* (AT5G44420), *VQ1* (AT1G17147), *VQ2* (AT1G21320), *VQ3* (AT1G21326), *VQ4* (AT1G28280), *VQ5* (AT1G32585), *VQ6* (At1G32610), *VQ7* (AT1G35830), *VQ8* (AT1G68450), *VQ9* (AT1G78310), *VQ10* (AT1G78410), *VQ11* (AT1G80450), *VQ13* (AT2G33780), *VQ14* (AT2G35230), *VQ15* (AT2G41010), *VQ16* (AT2G41180), *VQ17* (AT2G42140), *VQ18* (AT2G44340), *VQ19* (AT3G15300), *VQ20* (AT3G18360), *VQ21* (AT3G18690), *VQ22* (AT3G22160), *VQ23* (AT3G56710), *VQ24* (AT3G56880), *VQ25* (AT3G58000), *VQ26* (AT3G60090), *VQ27* (AT4G15120), *VQ28* (AT4G20000), *VQ30* (AT4G39720), *VQ31* (AT5G08480), *VQ32* (AT5G46780), *VQ33* (AT5G53830), *VQ34* (AT5G65170).

**How to cite this article**: Wang, H. *et al.* *Arabidopsis* VQ motif-containing proteins VQ12 and VQ29 negatively modulate basal defense against *Botrytis cinerea*. *Sci. Rep.* **5**, 14185; doi: 10.1038/srep14185 (2015).

## Supplementary Material

Supplementary Information
