# Supplementary material for: Arabidopsis VQ motif-containing proteins VQ12 and VQ29 negatively modulate basal defense against Botrytis cinerea
Source: Sci Rep. 2015 Sep 23;5:14185. doi: 10.1038/srep14185 (PMC4585807; doi:10.1038/srep14185)
Supplement: Supplementary Information [file srep14185-s1.pdf]

Arabidopsis VQ motif-containing proteins VQ12 and VQ29 negatively modulate basal defense against _Botrytis cinerea_

Houping Wang, Yanru Hu, Jinjing Pan, and Diqiu Yu

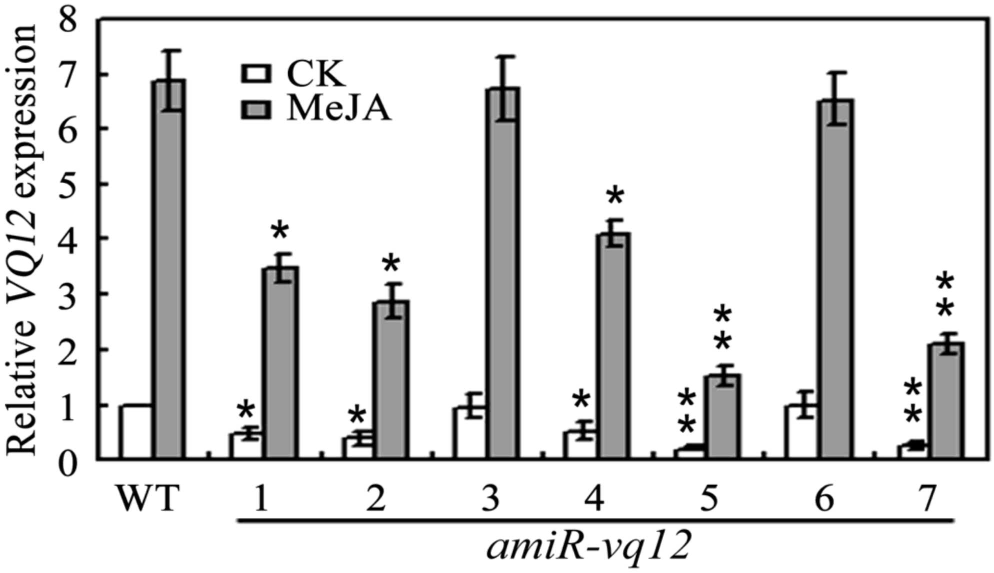

Figure S1. Analysis of *VQ12* expression levels in wild type (WT) and *amiR-vq12* transgenic plants. Total RNA was extracted from thirty-day-old WT or *amiR-vq12* after spraying with H_2O (CK) or MeJA (100 μM) for 3 h. Error bars indicate SD from three independent RNA extracts; statistics by Student's t test; *$p < 0.05$; **$p < 0.01$.

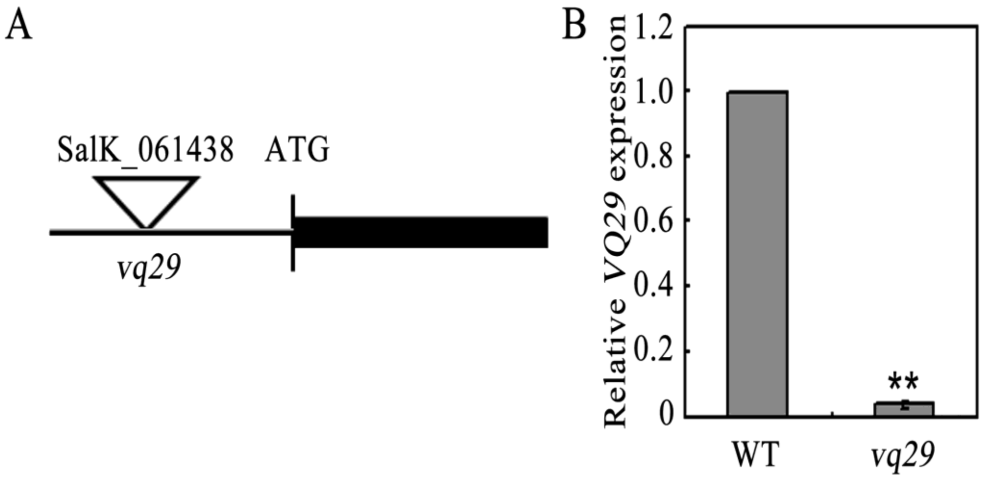

Figure S2. Isolation of *vq29* mutant. (A) Diagram of *VQ29* and the position of the T-DNA insertion. (B) qRT-PCR analyses of *VQ29* expression levels in wild type (WT) and *vq29* mutant. Total RNA was extracted from thirty-day-old WT or *vq29* without any treatment. Error bars indicate SD from three independent RNA extracts; statistics by Student's t test; **$p < 0.01$.

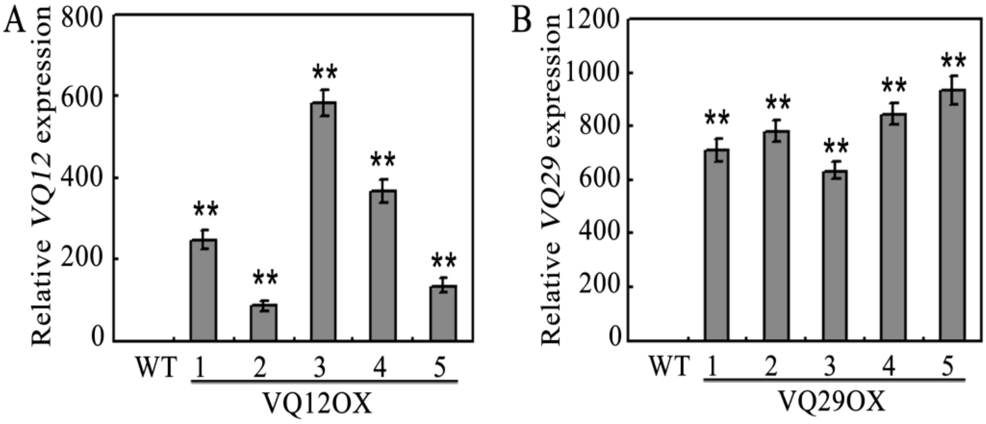

Figure S3. Over-expression lines for *VQ12* and *VQ29*. (A) Analysis of *VQ12* expression levels in wild type (WT) and VQ12OX transgenic plants. (B) Analysis of *VQ29* expression levels in WT and VQ29OX transgenic plants. Total RNA was extracted from thirty-day-old WT or transgenic plants without any treatment. Error bars indicate SD from three independent RNA extracts; statistics by Student's t test; **$p < 0.01$.

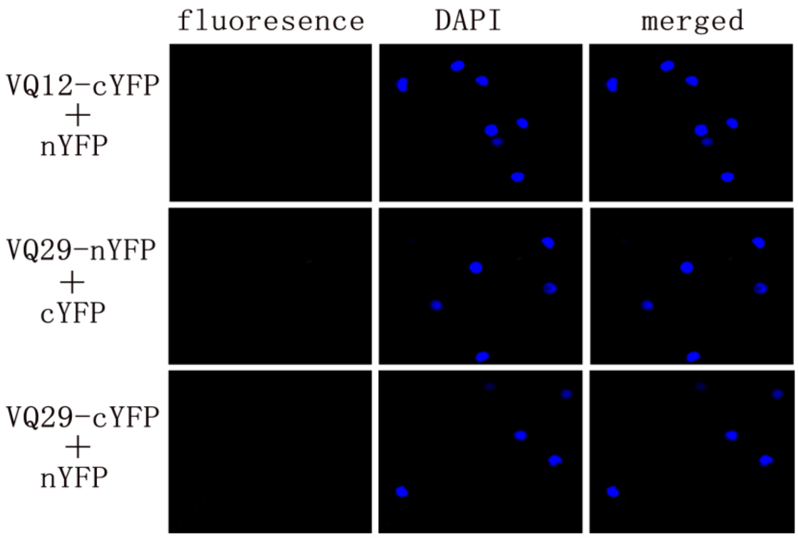

Figure S4. BiFC analysis. No signals were observed from the negative controls. DAPI staining marks the nucleus.

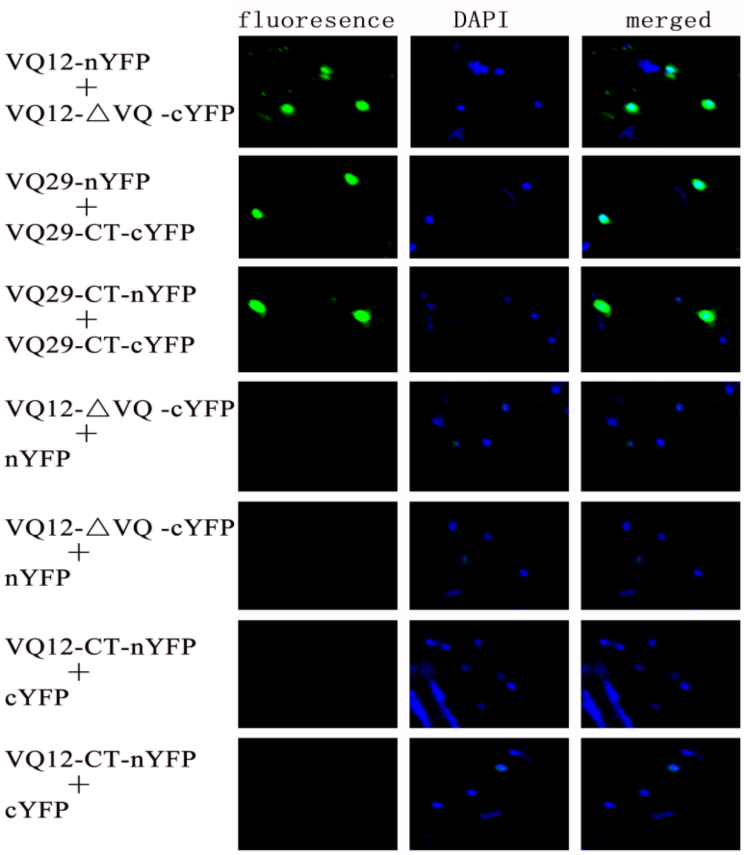

Figure S5. BiFC analysis. BiFC assay showing the fluorescence complementation of the N-terminal part of YFP fused with VQ12 or VQ29 and the C-terminal part of YFP fused with the C-terminal part of VQ29. No signals were observed from the negative controls. DAPI staining marks the nucleus.

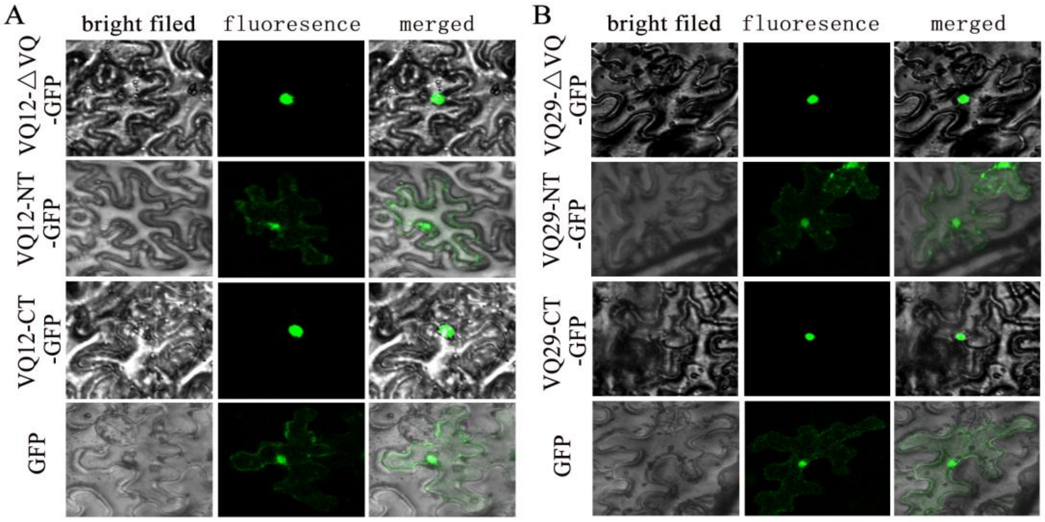

Figure S6. Subcellular localizations of the mutant forms of VQ12 and VQ29.

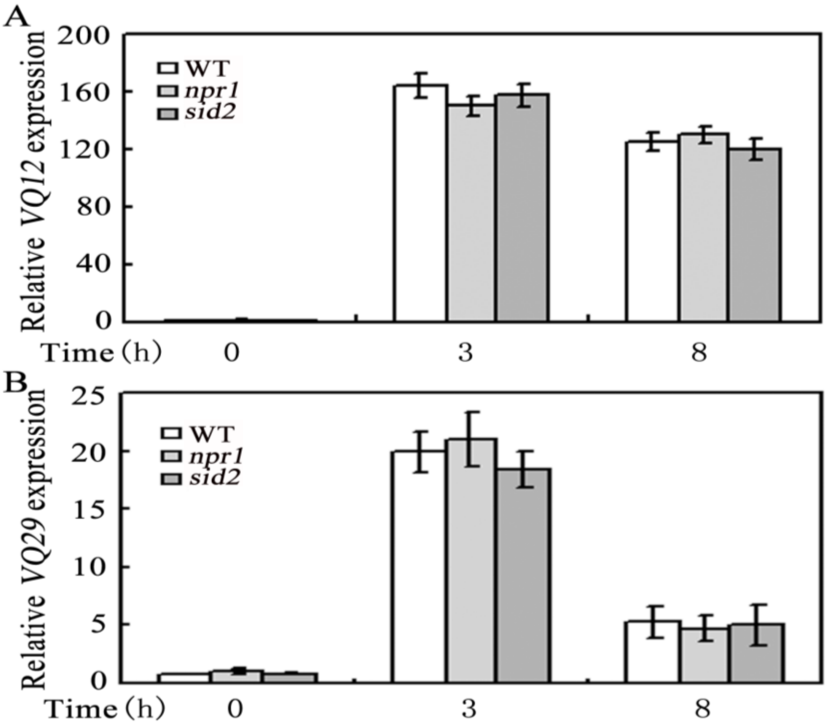

Figure S7. qRT-PCR analysis of *VQ12* (A) and *VQ29* (B) expression in response to *Pst*DC3000 infection in wild type (WT) and various defense-related mutants. Total RNA was extracted from thirty-day-old WT or mutant plants at 0, 1 and 2 dpi. Error bars indicate SD from three independent RNA extracts.

Table S1. Primers used in this study.

Use	Gene name	Primers (5'->3')
**qRT-PCR**	VQ12	qVQ12-F: TCACTTCATTCGACCCGTAAG<br>qVQ12-R: TGCACCAGTTAGCCTCTGAAC
	VQ29	qVQ29-F: CACAAGGGCAACGAAAAATTA<br>qVQ29-R: GACATGAGGATGCATTGGATT
	PDF1.2	qPDF1.2-F: TCACCCTTATCTTCGCTGCTCT<br>qPDF1.2-R: ATGATCCATGTTTGGCTCCTTC
	Thi2.1	qThi2.1-F: TTAGTAGTGTATTCTTTATACG<br>qThi2.1-R: TTCTACGACACATGCACAC
	PR4	qPR4-F: CGTGAGTGCTTATTGCTCCA<br>qPR4-R: ATACTTGCTCCGCCATGC
	β-*tubulin*	qβ-*tubulin*-F: TCTTGAGAGCGGTGGTATC<br>qβ-*tubulin*-R: TTGCATACGATCGGAGATACCT
**Yeast Two Hybrid**	VQ1	AD-VQ1-F: ATACCCGGGTATGTCTGCAGGAGTGAGATCTGA<br>AD-VQ1-R: ATAGGATCCATGGGCCTTAATATTGTGGGAA
	VQ2	AD-VQ2-F: ATAGAATTCATGGATAATAGATCGCCAAGATCAAGAG<br>AD-VQ2-R: ATAGGATCCTCACCTTCTTCCTCCACGCTGT
	VQ3	AD-VQ3-F: ATAGAATTCATGGATAATAGATCGCCAAGATCA<br>AD-VQ3-R: ATAGGATCCTCAAGAATCAAAGAAATTGTTGAAAAG
	VQ4	AD-VQ4-F: ATACCCGGGAATGGAGA ATTCACCGAGATACAGA<br>AD-VQ4-R: ATACTCGAGTCAAGAAGTAGAAGCTGATGAAGAACC
	VQ5	AD-VQ5-F: ATACCCGGGAATGTATCAGCGACCACAAAATG<br>AD-VQ5-R: ATAGAGCTCTCAATTTATTTCGTATGAGAATTCTAAATCC
	VQ6	AD-VQ6-F: ATACCCGGGGATGGATAGGACTTGTTGGTATTATGA<br>AD-VQ6-R: ATAGGATCCATGGAGCATCTTAGTAACCTCTCCA
	VQ7	AD-VQ7-F: ATACCCGGGCATGGATTCTTGTAACAGTGGAAGC<br>AD-VQ7-R: ATAGGATCCTTAATTATTGGAATCTGTAGGGCAAATC
	VQ8	AD-VQ8-F: ATAGAATTCATGATTCCAA CAAGATGCAA TGA<br>AD-VQ8-R: ATAGGATCCCTTATTCGGACTTAAAAACCAAGGG
	VQ9	AD-VQ9-F: ATAGAATTCATGGATAAGAGCTGTAACTCCTCC<br>AD-VQ9-R:ATACTCGAGCAAAACGACGACGTTTTCTCACT
	VQ10	AD-VQ10-F: ATAGAATTCATGTCTGGAAGAGGGAAAGTGAA<br>AD-VQ10-R: ATAGGATCCTCAATATTCTGACCATAGTTTATACAATTCTTC
	VQ11	AD-VQ11-F: ATAGAATTCATGAGTCACCAGCAGCCGCC<br>AD-VQ11-R: ATAGGATCCCTAAGAGTCTCGATGATTATCTTCATGAATCC
	VQ12	AD-VQ12-F: ATAGAATTCATGGAAGCTACTTCACAACCATGT<br>AD-VQ12-R: ATAGGATCCCTACCATCTTGATAGATTAGCAGGACTAA<br><br>BD-VQ12-F: ATAGAATTCGCAAAGCCATGGAAAAAACC<br>BD-VQ12-R: ATAGGATCCCTACCATCTTGATAGATTAGCAGGACTAA<br><br>BD-VQ12-CT-F: ATAGAATTCCTAACTGGTGCAGAAGACGTTGAG<br>BD-VQ12-CT-R: ATAGGATCCCTACCATCTTGATAGATTAGCAGGACTAA<br><br>BD-VQ12-NT-F: ATAGAATTCGCAAAGCCATGGAAAAAACC<br>BD-VQ12-NT-R: ATAGGATCCCCTCTGAACTAACTCCTTGAAG<br><br>BD-VQ12ΔVQ: ATAGAATTCGCAAAGCCATGGAAAAAACC<br>TTCCAGATCTTCCTCCTTGAAGTTAACAGGCTCGA<br>GAAGATCTGGAACTAACTGGTGCAGAAGACGTTGAG<br>ATAGGATCCCTACCATCTTGATAGATTAGCAGGACTAA

**Yeast Two Hybrid**		AD-VQ12-CT-F: ATAGAATTCCTAACTGGTGCAGAAGACGTTGAG<br>AD-VQ12-CT-R: ATAGGATCCCTACCATCTTGATAGATTAGCAGGACTAA
	VQ13	AD-VQ13-F : ATAGAATTCATGGAGAAATCACCAAGATACAGAGA<br>AD-VQ13-R: ATAGGATCCAGCAAAACTAGGGTTGATCAATGAT
	VQ14	AD-VQ14-R: ATAGAATTCATGGCTCCGCCACAGTCTCA<br>AD-VQ14-R: ATAGGATCCGCACCTAGTAATCATTCCATCTTGGAC
	VQ15	AD-VQ15-R: ATAGAATTCATGGTGACTTCGGAGGGATTAG<br>AD-VQ15-R: ATACTCGAGTCACATAACCTTCCACGATTCAA
	VQ16	AD-VQ16-F: ATAGAATTCATGGATCAGTCATCATCAACGTT<br>AD-VQ16-R: ATAGAGCTCTCAGTGCCGAAACAAAACATT
	VQ17	AD-VQ17-F: ATAGAATTCATGGA AATTGAAGCTACTACTGTTCAGA<br>AD-VQ17-R: ATAGGATCCTGAGACGTTTTAGGCGTAATGGT
	VQ18	AD-VQ18-F: ATACCCGGGAATGGAGATTACTCAATATCAAAGTTTTCA<br>AD-VQ18-R: ATAGGATCCGCAGAATCAATAAGATTGAAAACCC
	VQ19	AD-VQ19-F: ATAGAATTCATGGA GATTTCAACA AACCCAC<br>AD-VQ19-R: ATAGGATCCAGGTCTCTCAGTCTACATCTCCG
	VQ20	AD-VQ20-F: ATAGAATTCATGAGCTCAACGTACAAGGACAAC<br>AD-VQ20-R: ATAGGATCCTTAAAAATCGCGAAACTCCGT
	VQ21	AD-VQ21-F: ATAGAATTCATGGATCCG TCGGAGTATT TTG<br>AD-VQ21-R: ATACTCGAGACCCTTTGGGCTACTACATTGGA
	VQ22	AD-VQ22-F: ATAGAATTCATGGCTAACCCCAACGAGTG<br>AD-VQ22-R: ATACTCGAGTCCAATCGTTTTCTCATTGCAA
	VQ23	AD-VQ23-F: ATAGAATTCATGGAGTCATCATCGTCGACTT<br>AD-VQ23-R: ATAGGATCCATTATCACATAGAATCGATGCTTCCA
	VQ24	AD-VQ24-F: ATAGAATTCATGGCGTCGTCGGAGGGATTA<br>AD-VQ24-R: ATAGGATCCGACTCCATAGCTGCATAAGCTTAAGG
	VQ25	AD-VQ25-F: ATAGAATTCATGGAAGCCACGATCTTCGA<br>AD-VQ25-R: ATAGGATCCTTAAGCGAATTGATCTGAGAAAACTG
	VQ26	AD-VQ26-F: ATAGAATTCATGGTGAGAAATTCTATGAAGGCTG<br>AD-VQ26-R: ATAGGATCCCATATTAGGGTAAGGTGTGACCAAAC
	VQ27	AD-VQ27-F: ATAGAATTCATGGCCAACTCTAACAACGACTG<br>AD-VQ27-R: ATAGGATCCTCCTTTATGTTTTTTTCCTCGGA
	VQ28	AD-VQ28-F: ATAGAATTCATGAACAACTCTAGAGAAGACCAGTG<br>AD-VQ28-R: ATAGGATCCTCCCCTAAGTAACATTATGATGATCA
	VQ29	AD-VQ29-F: ATAGAATTCATGGAAGCAACATCACAACAATT<br>AD-VQ29-R: ATAGGATCCAGCTACCATCTGGAAATATTTGCA<br><br>BD-VQ29-F: ATAGAATTCAGGGCAACGAAAAATTACCTAACT<br>BD-VQ29-R: ATAGGATCCCTACCATCTGGAAATATTTGCAGG<br><br>BD-VQ29-CT-F: ATAGAATTCCTAACCGGTGCACCTGAGCA<br>BD-VQ12-CT-R: ATAGGATCCCTACCATCTGGAAATATTTGCAGG<br><br>BD-VQ29-NT-F: ATAGAATTCAGGGCAACGAAAAATTACCTAACT<br>BD-VQ29-NT-R: ATAGGATCCCCTCTGAACCAACACTTTGAAGTT<br><br>BD-VQ29ΔVQ: ATAGAATTCAGGGCAACGAAAAATTACCTAACT<br>TTCCAGATCTTCCACTTTGAAGTTTACTGGCTCGAC<br>GAAGATCTGGAACTAACCGGTGCACCTGAGCA<br>ATAGGATCCCTACCATCTGGAAATATTTGCAGG<br><br>AD-VQ29-CT-F: ATAGAATTCCTAACCGGTGCACCTGAGCA

Yeast Two Hybrid		AD-VQ12-CT-R: ATAGGATCCCTACCATCTGGAAATATTTGCAGG
	VQ30	AD-VQ30-F: ATAGAATTCATGGAGTCCGGTAATAGTAGTAGCATG<br>AD-VQ30-R:ATAGGATCCTGTTCTCTGGTCGGAAGACG
	VQ31	AD-VQ31-F: ATAGAATTCATGAATAGCA AAGGGAGTCA AAAC<br>AD-VQ31-R: ATAGGATCCTCATGGTTTGCCACTCGAAT
	VQ32	AD-VQ32-F: ATAGAATTCATGGATGATCAGAGTAATCGTGGT<br>AD-VQ32-R: ATAGGATCCTGGAGCACTTAATCCACCATC
	VQ33	AD-VQ33-F: ATAGAATTCATGGAAGTTTCAACATCATCCATG<br>AD-VQ33-R: ATAGGATCCACTCATGGATATCGTCGGGATT
	VQ34	AD-VQ34-F: ATACCCGGGCATGGAATCCGGCAATAGTAGTAGC<br>AD-VQ34-R: ATAGAGCTCTGCATCTAATCAGAAGAAGAGATCCAA
	WRKY33	BD-WRKY33-CT-F: ATCGAATTCGTAGTGCAGACAACGAGTGAT<br>BD-WRKY33-CT-R: ATCGTCGACTCAGGGCATAAACGAATCGA
BiFC	VQ12	YN-VQ12-F: ATAGAGCTCATGGAAGCTACTTCACAACCATGT<br>YN-VQ12-R: ATAGGATCCCCATCTTGATAGATTAGCAGGACTAA<br><br>YC-VQ12-F: ATAGAGCTCATGGAAGCTACTTCACAACCATGT<br>YC-VQ12-R: ATAGGATCCCCATCTTGATAGATTAGCAGGACTAA<br><br>YN-VQ12-CT-F: ATAGAGCTCCTAACTGGTGCAGAAGACGTTGAG<br>YN-VQ12-CT-R: ATAGGATCCCTACCATCTTGATAGATTAGCAGGACTAA<br><br>YN-VQ12ΔVQ: ATAGAGCTCGCAAAGCCATGGAAAAAACC<br>TTCCAGATCTTCCTCCTTGAAGTTAACAGGCTCGA<br>GAAGATCTGGAACTAACTGGTGCAGAAGACGTTGAG<br>ATAGGATCCCTACCATCTTGATAGATTAGCAGGACTAA<br><br>YC-VQ12-CT-F: ATAGAGCTCCTAACTGGTGCAGAAGACGTTGAG<br>YC-VQ12-CT-R: ATAGGATCCCTACCATCTTGATAGATTAGCAGGACTAA<br><br>YC-VQ12ΔVQ: ATAGAGCTCGCAAAGCCATGGAAAAAACC<br>TTCCAGATCTTCCTCCTTGAAGTTAACAGGCTCGA<br>GAAGATCTGGAACTAACTGGTGCAGAAGACGTTGAG<br>ATAGGATCCCTACCATCTTGATAGATTAGCAGGACTAA
	VQ29	YN-VQ29-F: ATAGAGCTCATGGAAGCAACATCACAACAATT<br>YN-VQ29-R: ATAGGATCCCCATCTGGAAATATTTGCAGGA<br><br>YC-VQ29-F: ATAGAGCTCATGGAAGCAACATCACAACAATT<br>YC-VQ29-R: ATAGGATCCCCATCTGGAAATATTTGCAGGA<br><br>YN-VQ29-CT-F: ATAGAGCTCCTAACCGGTGCACCTGAGCA<br>YN-VQ12-CT-R: ATAGGATCCCTACCATCTGGAAATATTTGCAGG<br><br>YN-VQ29ΔVQ: ATAGAGCTCAGGGCAACGAAAAATTACCTAACT<br>TTCCAGATCTTCCACTTTGAAGTTTACTGGCTCGAC<br>GAAGATCTGGAACTAACCGGTGCACCTGAGCA<br>ATAGGATCCCTACCATCTGGAAATATTTGCAGG<br><br>YC-VQ29-CT-F: ATAGAGCTCCTAACCGGTGCACCTGAGCA<br>YC-VQ12-CT-R: ATAGGATCCCTACCATCTGGAAATATTTGCAGG<br><br>YC-VQ29ΔVQ: ATAGAGCTCAGGGCAACGAAAAATTACCTAACT<br>TTCCAGATCTTCCACTTTGAAGTTTACTGGCTCGAC<br>GAAGATCTGGAACTAACCGGTGCACCTGAGCA<br>ATAGGATCCCTACCATCTGGAAATATTTGCAGG
Transgenic Plants	VQ12	amiRvq12-F:ATGATATCGAaTCGCGCCTGTaAACTTCtTCACAGGTCGTGATAT<br>GATTCA<br>amiRvq12-R:AAGAATTCGAGTCGCGCCTGTTAACTTCATCAAAGAGAATCAA<br>TGATCCA

**Transgenic Plants**	 <br><br>VQ29	OXVQ12- F: ATAGAGCTCATGGAAGCTACTTCACAACCATGTT<br>OXVQ12- R: ATAGGATCCCTACCATCTTGATAGATTAGCAGGACTAA<br><br>OXVQ29- F: ATAGAGCTCATGGAAGCAACATCACAACAATTTA<br>OXVQ29- R: ATAGGATCCCTACCATCTGGAAATATTTGCAGG
**Subcellular Localization**	VQ12<br><br><br><br><br><br><br><br><br><br><br><br><br><br>VQ29	GFP-VQ12-F: ATAGAGCTCATGGAAGCTACTTCACAACCATGT<br>GFP-VQ12-R: ATATCTAGACCATCTTGATAGATTAGCAGGACTAAG<br><br>GFP-VQ12-CT-F: ATAGAGCTCATGCTAACTGGTGCAGAAGACGTTGAG<br>GFP-VQ12-CT-R: ATATCTAGACTACCATCTTGATAGATTAGCAGGACTAA<br><br>GFP-VQ12-NT-F: ATAGAGCTCGCAAAGCCATGGAAAAAACC<br>GFP-VQ12-NT-R: ATATCTAGACCTCTGAACTAACTCCTTGAAG<br><br>GFP-VQ12ΔVQ: ATAGAGCTCGCAAAGCCATGGAAAAAACC<br>TTCCAGATCTTCCTCCTTGAAGTTAACAGGCTCGA<br>GAAGATCTGGAACTAACTGGTGCAGAAGACGTTGAG<br>ATATCTAGACTACCATCTTGATAGATTAGCAGGACTAA<br><br>GFP-VQ29-F: ATAGAGCTCATGGAAGCAACATCACAACAATT<br>GFP-VQ29-R: ATAGGATCCCCATCTGGAAATATTTGCAGGA<br><br>GFP-VQ29-CT-F: ATAGAGCTCATGCTAACCGGTGCACCTGAGCA<br>GFP-VQ12-CT-R: ATAGGATCCCTACCATCTGGAAATATTTGCAGG<br><br>GFP-VQ29-NT-F: ATAGAGCTCAGGGCAACGAAAAATTACCTAACT<br>GFP-VQ29-NT-R: ATAGGATCCCCTCTGAACCAACACTTTGAAGTT<br><br>GFP-VQ29ΔVQ: ATAGAGCTCAGGGCAACGAAAAATTACCTAACT<br>TTCCAGATCTTCCACTTTGAAGTTTACTGGCTCGAC<br>GAAGATCTGGAACTAACCGGTGCACCTGAGCA<br>ATAGGATCCCTACCATCTGGAAATATTTGCAGG
